# Pre-participation screening of asymptomatic athletes: “Don’t do stupid stuff”

**DOI:** 10.1007/s12471-018-1075-7

**Published:** 2018-02-01

**Authors:** A. Mosterd

**Affiliations:** Department of Cardiology, Meander Medical Center, Amersfoort, The Netherlands

**Keywords:** Athletes, Cardiovascular, Screening, Sudden cardiac death

## Abstract

Catastrophic events, be it traffic accidents, natural disasters or homicides, always lead to scrutiny. Could we have seen the event coming and could it have been prevented? In the case of a sudden cardiac arrest of a seemingly healthy athlete the public outcry is not any different. Despite an intrinsic appeal for screening to prevent similar events, there is no evidence that justifies routine cardiovascular pre-participation screening of athletes. On balance, cardiovascular screening in athletes will most likely do more harm than good. Fatal exercise-related cardiac arrests do not occur very often. The true diagnostic yield of the pre-participation evaluation is not known and once a cardiac condition has been identified, the most appropriate intervention is often unclear. It follows that pre-participation screening of large groups of athletes without known cardiac disease will inevitably result in many false positive findings, while at the same time providing a false sense of security to those screened negative. Except for compelling reasons (e. g. cascade screening, research settings, professional athletes), physicians should not engage in routine examination of asymptomatic athletes to prevent cardiac events.

## Introduction

Catastrophic events, be it traffic accidents, natural disasters or homicides, always lead to scrutiny. Could we have seen the event coming and could it have been prevented? In the case of a sudden cardiac arrest of a seemingly healthy athlete the public outcry is not any different.

Despite an intrinsic appeal for screening to prevent similar events, the sobering 2006 conclusion by the Health Council of the Netherlands, stating there is no evidence that justifies routine cardiovascular pre-participation screening in young athletes, still stands [1]. As a matter of fact, there is increasing evidence supporting the appeal to steer away from routine pre-participation screening [2–6]. Recent recommendations from the UK’s national screening committee, as well as from the American Heart Association, advise against screening [7, 8].

In this point of view, I build on earlier commentaries and provide a brief overview of recent developments in the field of the cardiovascular evaluation of athletes, notably the extensive review of the literature undertaken by the Belgian Health Care Knowledge Centre [2, 9, 10].

### A brief history of cardiovascular pre-participation screening

Italian experience with mandatory pre-participation screening since the 1980s formed the basis for the 2005 European Society of Cardiology (ESC) consensus statement on pre-participation screening of young athletes (aged 12–35 years) [11, 12]. The recommended evaluation to prevent exercise-related cardiac arrests consists of a personal and family medical history, a physical examination and a 12-lead resting electrocardiogram. The ESC statement has led to a heated debate between proponents and opponents of mandatory pre-participation screening [13, 14].

It is important that we realise that the ESC statement is a proposal for a common European protocol rather than a guideline with an imperative to screen. Guideline recommendations are preferably based on randomised controlled trials. We have no such trials on the effects of pre-participation screening and given the sheer size of a trial required to address this issue (the Dutch National Institute for Public Health and the Environment calculated that a trial would involve two groups with at least 1,200,000 person-years follow-up), a randomised evaluation will probably never see the light of day [15].

Observational data, without a contemporary control group of non-screened athletes, from the Veneto region showed a decrease in the incidence of sudden cardiac death in young athletes in the 1980s that was attributed to pre-participation screening [11]. In contrast, a nationwide study in Israel could not demonstrate a decline in the incidence of sudden cardiac death in athletes after the introduction of mandatory screening in the mid-1990s [3]. The very recent Canadian study on sudden cardiac arrests, conducted from 2009 through 2014 in athletes, competitive and non-competitive, aged 12–45 years, reported that pre-participation screening would have missed more than 80% of cases [5].

The main findings of an extensive literature review of pre-participation screening in young athletes commissioned by the Belgian government were summarised in the British Medical Journal in April 2016 [2]:

- An estimated 0.001% of young athletes die suddenlyevery year
- Up to 30% of those screened may be referred for additional testing
- Screening would not detect around 25% of those at risk of sudden death
- On balance, cardiovascular screening in young athletes is likely to be more harmful than beneficial.

Taken all together, it does not come as a surprise that the latest (2017) ESC statement strikes a more cautious tone in relation to the benefits of pre-participation screening (‘It goes far beyond the scope of the document to suggest global national pre-participation evaluation programmes’) [16].

### Pitfalls of screening

Screening programmes generally do not live up to expectations and the real implications of screening are not appreciated by the majority of physicians, let alone the lay public [17]. A successful screening programme cost-effectively addresses a common condition (A), the presence of which can reliably be diagnosed with an affordable test (B) which subsequently will predictably result in adverse events that can be prevented by an accepted intervention (C).

A. Fatal exercise-related cardiac arrests do not occur very often, less than 1 per 100,000 per year in those aged 35 years or younger and five to ten times more frequently in those older than 35 years, with an overwhelming male preponderance (10:1) [4–6]. The cause of cardiac arrest in athletes aged 35 years or younger is a mixed bag of inherited cardiac disease (cardiomyopathies, electrical heart disease), coronary anomalies, myocarditis, blunt chest trauma and coronary artery disease. In older athletes, coronary artery disease is the main cause [4, 6].
B. The true diagnostic yield/accuracy of the proposed cardiovascular pre-participation evaluation, essentially consisting of a medical history, physical examination and the resting 12-lead electrocardiogram as a cornerstone, is not known. Coronary anomalies and premature coronary artery disease, for example, will not be found with this approach and even a seemingly straightforward electrocardiogram diagnosis of long QT syndrome is known to be missed by experienced cardiologists [18]. The fact that exercise per se results in electrocardiogram changes complicates matters even more, notwithstanding efforts to standardise and improve the electrocardiogram analysis of athletes [19].
C. Once a cardiac condition has been identified, the next challenge presents itself; should we advise restricting sporting activities and should we otherwise intervene in those who are truly asymptomatic [20]? With regard to the former: many athletes decide to continue their activities, which recently led to a plea to strike a less paternalistic tone in the counselling of athletes with cardiovascular disease [21]. With regard to the latter: at least two studies demonstrated that lifelong intensive exercisers are likely to have coronary atherosclerosis more often than their counterparts with lower lifelong exercise volumes [22, 23]. However, the most active athletes showed a more benign pattern of coronary atherosclerosis; less mixed plaques and more often only calcified plaques. Currently, we have no guidance on how to treat these athletes, if at all. The presence of coronary atherosclerosis is associated with a worse outcome in the general population, but in athletes who have exercised intensively for many years this association may be altered given the more benign aspect of their atherosclerotic plaques.

It follows that routine pre-participation screening of large groups of athletes without known cardiac disease will inevitably result in a large number of false positive findings (leading to substantial psychological distress and costly additional investigations—just to be sure—, not to mention insurance issues) while at the same time providing a false sense of security in those who screened negative.

### Alternatives to screening

Accepting the fact that timely identification of athletes at increased risk of sudden cardiac death is currently not possible, what other options do we have? Studies from Canada, the Netherlands and France have convincingly shown that exercise-related cardiac arrest is becoming a treatable condition (with nearly 50% of victims surviving in areas with an adequate chain of resuscitation actions) [5, 6, 24]. Prompt bystander resuscitation efforts with the use of increasingly available automated external defibrillators have resulted in increased survival rates, not restricted to athletes [25].

A solid infrastructure for cardiogenetic evaluation (including cascade screening, i. e. the systematic testing of relatives of a known mutation carrier to identify those who might benefit from an intervention) of persons with inherited cardiac disease may help timely identification of those at an increased risk of adverse cardiovascular events.

### “*First, do no harm*”

Except for compelling reasons (e. g. cascade screening, research settings, professional athletes), physicians should not engage in routine examination of asymptomatic athletes to prevent cardiac events. To quote former US president Barack Obama: “Don’t do stupid stuff”.

## References

[1] Health council of the Netherlands (2006). Jaarbericht Bevolkingsonderzoek.

[2] Van Brabandt H, Desomer A, Gerkens S, Neyt M (2016). Harms and benefits of screening young people to prevent sudden cardiac death. BMJ.

[3] Steinvil A, Chundadze T, Zeltser D (2011). Mandatory electrocardiographic screening of athletes to reduce their risk for sudden death proven fact or wishful thinking?. J Am Coll Cardiol.

[4] Marijon E, Tafflet M, Celermajer DS (2011). Sports-related sudden death in the general population. Circulation.

[5] Landry CH, Allan KS, Connelly KA (2017). Sudden cardiac arrest during participation in competitive sports. N Engl J Med.

[6] Berdowski J, de Beus MF, Blom M (2013). Exercise-related out-of-hospital cardiac arrest in the general population: incidence and prognosis. Eur Heart J.

[7] UK National Screening Committee (2015). The UK NSC recommendation on screening to prevent sudden cardiac death in 12 to 39 year olds.

[8] Maron BJ, Levine BD, Washington RL, American Heart Association Electrocardiography and Arrhythmias Committee of Council on Clinical Cardiology, Council on Cardiovascular Disease in Young, Council on Cardiovascular and Stroke Nursing, Council on Functional Genomics and Translational Biology, American College of Cardiology (2015). Eligibility and disqualification recommendations for competitive athletes with cardiovascular abnormalities: task force 2: preparticipation screening for cardiovascular disease in competitive athletes: a scientific statement from the American Heart Association and American College of Cardiology. Circulation.

[9] Mosterd A, Senden JP, Engelfriet P (2011). Preventing sudden cardiac death in athletes: finding the needle in the haystack or closing the barn door?. Eur J Cardiovasc Prev Rehabil.

[10] Mosterd A, Velthuis BK (2012). Screening of athletes is undesirable. Ned Tijdschr Geneeskd.

[11] Corrado D, Basso C, Pavei A (2006). Trends in sudden cardiovascular death in young competitive athletes after implementation of a preparticipation screening program. JAMA.

[12] Corrado D, Pelliccia A, Bjornstad HH (2005). Cardiovascular pre-participation screening of young competitive athletes for prevention of sudden death: proposal for a common European protocol. Consensus statement of the Study Group of Sport Cardiology of the Working Group of Cardiac Rehabilitation and Exercise Physiology and the Working Group of Myocardial and Pericardial Diseases of the European Society of Cardiology. Eur Heart J.

[13] Viskin S (2007). Antagonist: routine screening of all athletes prior to participation in competitive sports should be mandatory to prevent sudden cardiac death. Heart Rhythm.

[14] Corrado D, Thiene G (2007). Protagonist: routine screening of all athletes prior to participation in competitive sports should be mandatory to prevent sudden cardiac death. Heart Rhythm.

[15] Engelfriet PM, van Gils PF, Smit HA (2009). Preparticipation screening in order to prevent sudden cardiac death: ‘Italian design’ for Dutch athletes?.

[16] Mont L, Pelliccia A, Sharma S (2017). Pre-participation cardiovascular evaluation for athletic participants to prevent sudden death: position paper from the EHRA and the EACPR, branches of the ESC. Eur J Prev Cardiol.

[17] Wegwarth O, Schwartz LM, Woloshin S (2012). Do physicians understand cancer screening statistics? A national survey of primary care physicians in the United States. Ann Intern Med.

[18] Viskin S, Rosovski U, Sands AJ (2005). Inaccurate electrocardiographic interpretation of long QT: the majority of physicians cannot recognize a long QT when they see one. Heart Rhythm.

[19] Sharma S, Drezner JA, Baggish A (2017). International recommendations for electrocardiographic interpretation in athletes. J Am Coll Cardiol.

[20] Maron BJ, Zipes DP, Kovacs RJ, American Heart Association Electrocardiography, Arrhythmias Committee of the Council on Clinical Cardiology, Council on Cardiovascular Disease in the Young, Council on Cardiovascular and Stroke Nursing, Council on Functional Genomics and Translational Biology, American College of Cardiology, American College of Cardiology (2015). Eligibility and disqualification recommendations for competitive athletes with cardiovascular abnormalities: preamble, principles, and general considerations: a scientific statement from the American Heart Association and American College of Cardiology. Circulation.

[21] Baggish AL, Ackerman MJ, Lampert R (2017). Competitive sport participation among athletes with heart disease: a call for a paradigm shift in decision making. Circulation.

[22] Aengevaeren VL, Mosterd A, Braber TL (2017). Relationship between lifelong exercise volume and coronary atherosclerosis in athletes. Circulation.

[23] Merghani A, Maestrini V, Rosmini S (2017). Prevalence of subclinical coronary artery disease in masters endurance athletes with a low atherosclerotic risk profile. Circulation.

[24] Marijon E, Bougouin W, Celermajer DS (2013). Major regional disparities in outcomes after sudden cardiac arrest during sports. Eur Heart J.

[25] Blom MT, Beesems SG, Homma PC (2014). Improved survival after out-of-hospital cardiac arrest and use of automated external defibrillators. Circulation.

